# Mini-Review: The Contribution of Adipokines to Joint Inflammation in Inflammatory Rheumatic Diseases

**DOI:** 10.3389/fendo.2020.606560

**Published:** 2020-12-23

**Authors:** Eric Toussirot

**Affiliations:** ^1^ INSERM CIC-1431, Centre d’Investigation Clinique Biothérapie, Pôle Recherche, CHU de Besançon, Besançon, France; ^2^ Fédération Hospitalo-Universitaire INCREASE, CHU de Besançon, Besançon, France; ^3^ Rhumatologie, Pôle PACTE (Pathologies Aiguës Chroniques Transplantation Éducation), CHU de Besançon, Besançon, France; ^4^ Département Universitaire de Thérapeutique, Université de Bourgogne Franche-Comté, Besançon, France; ^5^ INSERM UMR1098 « Relations Hôte Greffon Tumeurs, Ingénierie Cellulaire et Génique », Université de Bourgogne Franche-Comté, Besançon, France

**Keywords:** adipokines, rheumatoid arthritis, spondyloarthritis, inflammation, structural damage

## Abstract

Inflammatory rheumatic diseases (IRD) are complex disorders characterized by chronic inflammation of the joints and related skeletal structures. The most common forms of IRD are rheumatoid arthritis (RA) and spondyloarthritis (SpA), including axial SpA (axSpA) and psoriatic arthritis (PsA). Obesity is a frequent comorbidity in RA and PsA, and to a lesser extend in axial SpA. The association between obesity and IRD may be explained by the release from fat tissue of several bioactive proteins, namely adipokines. Adipokines are involved in the regulation of various processes such as lipid or glucose metabolism, but also inflammation. Adipokines are interrelated with the immune system, with both innate and adaptive immune cell connections. Several adipokines with pro-inflammatory effects have been identified such as leptin, visfatin or resistin. Conversely, adiponectin and more specifically its low molecular weight isoform, is considered to have antiinflammatory properties. In this review, we discuss the contribution of adipokines to the joint inflammation of IRD, the relation they have with immune pathways of these diseases, their links with the structural impact on peripheral joints and/or axial skeleton, and also the influence they may have on the cardiometabolic risk of IRD.

## Introduction

Inflammatory rheumatic diseases (IRD) are complex systemic disorders characterized by chronic inflammation of the joints and related musculo-skeletal components. They comprise diverse conditions of which rheumatoid arthritis (RA) and spondyloarthritis (SpA) are the most common.

Rheumatoid arthritis (RA) is a systemic immune-mediated disease with chronic symmetrical joint inflammation, joint deformations, erosive radiographic changes and disability. Pro-inflammatory cytokines such as IL-1β, TNFα, and IL-6, but also IL-17A and specific chemokines, have been identified as major players in the joint inflammation of RA (1). Spondyloarthritis (SpA) corresponds to a group of inflammatory disorders that mainly affect the axial skeleton (the spine and the sacroiliac joints), but also entheseal structures. Spondyloarthritis may be subclassified as axial (axSpA) or peripheral disease according to the main skeletal involvement. The most common forms of SpA are ankylosing spondylitis (AS) and psoriatic arthritis (PsA). Radiographic changes of the sacroiliac joints and ligamentous ossifications of the spine are specific hallmarks of axSpA (2). TNFα and IL-17A have been identified as key mediators driving inflammation in axSpA, while IL-23 and IL-17A, together with TNFα, are involved in the inflammatory process of PsA (3).

Specific comorbidities may be observed in RA and SpA. Indeed, it is well recognized that the development of atherosclerotic cardiovascular (CV) disease is a leading comorbidity of RA (4). Like RA, axSpA and PsA are also characterized by a high mortality rate linked to CV comorbidities (5).

Obesity is considered to be a systemic disease leading to several complications including metabolic and CV diseases, but also to the development of inflammatory diseases. Indeed, obesity is described to be highly prevalent in IRD and plays a role in their development (6, 7). The link between obesity, IRD and inflammation could be explained by the release from fat tissue of bioactive proteins, namely adipokines. The term adipokines refers to a variety of biological products that are mainly (but not exclusively) produced by the white adipose tissue (8, 9). For two decades, adipokines have drawn attention from the scientific community as potential mediators playing a role in the modulation of the immune system and inflammatory response (10–13).

In this review, we discuss the role and contribution of adipokines to the joint inflammation of IRD, with special emphasis on the more widely studied molecules in this field, *i.e.* leptin, adiponectin, visfatin, and resistin.

## Biological Properties of Leptin, Adiponectin, Visfatin, And Resistin

Adipokines are mainly produced by the adipocytes, but other cellular sources may synthetize these mediators, including immune cells or, at the joint level, synoviocytes or chondrocytes. They have autocrine, paracrine and endocrine actions on targets cells and organs such as the bone, cartilage and synovial membrane (8, 14). In IRD, high levels of adipokines have been described in both the blood circulation and the synovial fluid. The expression of certain adipokines has also been reported in the synovial membrane (15). Adipokines are interrelated with the immune system, both the innate and adaptive systems, underlying the role these mediators may play in IRD (9, 16, 17).

### Leptin

Leptin is a 16 kDa cytokine-like hormone that is mainly produced by white adipose tissue. Its primary function is to regulate appetite and energy balance by inducing anorexigenic factors and in turn, suppressing orexigenic neuropeptides in the hypothalamus. However, leptin has a wide spectrum of other physiological functions (18). The production of leptin is dependent on metabolic and energetic factors, including insulin and sex hormones, but pro-inflammatory mediators such as TNFα, IL-6 or IL-1β may also stimulate its release. Leptin is considered to be a pro-inflammatory adipokine by its interaction with the innate and adaptive immune system. Indeed, leptin upregulates the production of pro-inflammatory cytokines such as TNFα, IL-6 and IL-12, and in turn, TNFα and IL-1β increase the expression of leptin in adipose tissue (19). The administration of inflammatory stimuli such as lipopolysaccharide (LPS) increases the level or expression of leptin in serum or adipose tissue (20). Leptin can stimulate monocytes, macrophages, dendritic cells, neutrophils and NK cells (14). Leptin enhances the phagocytic activity of monocytes/macrophages and induces the production of eicosanoids, nitric oxide and several cytokines (21). In adaptive immunity, leptin promotes T cell activation and differentiation toward a Th1 polarization and suppresses a Th2 phenotype, leading to increased production of IFNγ and IL-2, and decreased production of IL-4 (22). Leptin is also associated with an increase in Th17 cell proliferation and reactivity (23). In parallel, leptin inhibits the production of regulatory T cells (24). Leptin may also promote cartilage degradation by increasing metalloprotease (MMP) and cysteine proteases, but also the production of IL-1β, TNFα, IL-6, IL-8 and monocyte chemoattractant protein 1 (MCP-1) by chondrocytes (25).

### Adiponectin

Adiponectin is mainly produced by the adipose tissue and exists in different molecular isoforms: globular adiponectin, full length adiponectin, and low (LMW), middle and high molecular weight (HMW) adiponectin (14). Adiponectin has a primarily metabolic function, by sensitizing to the action of insulin. Low levels of adiponectin are associated with insulin resistance and type 2 diabetes (T2D), but also dyslipidemia and hypertension (11, 14). Adiponectin is considered to have anti-inflammatory effects (26). However, the influence of adiponectin on the immune system and the inflammatory response is complex and depends on its different isoforms (12, 14). In fact, it is acknowledged that predominantly anti-inflammatory and beneficial effects of adiponectin have been described in atherosclerosis, metabolic syndrome and T2D (26). These favorable effects on the CV risk have been related to the HMW isoform of adiponectin. In parallel, opposite but contradictory effects have been reported in inflammatory conditions such as IRD (11). Adiponectin can suppress maturation and growth of macrophages in response to LPS. Adiponectin is able to induce the production of anti-inflammatory cytokines such as IL-10 and IL-1 receptor antagonist (27). Pro-inflammatory cytokines such as TNFα and IL-6 may inhibit adiponectin gene expression and protein release. Conversely, there is now some evidence that the different isoforms of adiponectin exert distinct and sometimes counteracting biological functions: LMW adiponectin inhibits LPS-mediated IL-6 release and stimulates IL-10 secretion, while HMW adiponectin induces the secretion of IL-6 by monocytes (14, 28). Increased production of pro-inflammatory mediators by cultured synovial fibroblasts has been observed in patients with RA in the presence of adiponectin (14). HMW adiponectin is able to induce the production of MCP-1 and IL-8 by peripheral blood mononuclear cells (PBMCs) (29). Collectively, it is considered that HMW adiponectin drives pro-inflammatory effects on the joint while the LMW isoform is associated with anti-inflammatory properties (11, 12, 14). However, this bidirectional effect of adiponectin depends on the relative proportion of its different isoforms, the cytokine milieu, and also the target cell or tissue that is studied (12). At the cartilage level, it has been demonstrated that adiponectin have an inflammatory profile, leading to the production by the chondrocytes of several pro-inflammatory mediators including IL-6, IL-8, but also nitric oxide (NO), vascular endothelial growth factor (VEGF), MCP-1, vascular cell adhesion protein-1 (VCAM-1) and different metalloproteases (MMP-1,MMP-3 and MMP-13) (30–32).

### Resistin

Resistin is a 12.5 kDa cysteine-rich protein that circulates in the blood as a homodimer. It is mainly produced by mononuclear cells and has been associated with inflammatory response by promoting immune cell activation (8). Resistin is also produced by adipocytes but in small amounts. Resistin is involved in different metabolic functions, including insulin resistance. High levels of circulating resistin correlate with overweight and other components of the metabolic syndrome (33). Resistin is found in areas of inflammation and is considered to be an important link between obesity and inflammation. Indeed, resistin is capable of inducing the production of IL-6, TNFα, and IL-1β by PBMCs (34). Synovial cells may also produce IL-6 and TNFα under the stimulation of resistin (35).

### Visfatin

Visfatin (or pre-B cell colony-enhancing factor or nicotinamide phosphoribosyltransferase) is mainly produced by visceral adipose tissue, an area of fat tissue whose accumulation strongly correlates with an enhanced CV risk. Visfatin is produced by the adipose tissue and has insulin-like effects (8). It regulates insulin secretion and insulin cellular signaling. Visfatin is able to produce pro-inflammatory effects in various cells through the release of TNFα, IL-1β, IL-6 and chemokines (11). Visfatin is associated with the production of chemokines, matrix-degrading factors and pro-angiogenic factors by synovial fibroblasts from patients with RA (36, 37).

## Adipokines in Rheumatoid Arthritis

Adipokines have been extensively studied in patients with RA, both at the systemic and local joint level. Results are available for leptin and adiponectin, and, to a lesser extent, for visfatin and resistin (8–13).

### Leptin

There are numerous studies showing elevated concentrations of leptin in the blood compartment of patients with RA compared to control populations (38–44). Correction of leptin levels for BMI or fat mass was not systematically performed, but certain studies still confirmed elevated concentrations of leptin independently of BMI (42). Since leptin has pro-inflammatory properties inducing the activation of both innate and adaptive immune cells, its relationships with markers of disease activity have also been analyzed. Collectively, the results showed that leptin correlated with CRP or disease activity score 28-joint (DAS28), but these relationships were not confirmed by all reports (11). Leptin was also measured in the synovial fluid and the concentration in the joint cavity was found to be higher in RA compared to patients with osteoarthritis (OA) (39). A meta-analysis concluded that leptin levels are higher in RA patients than in healthy controls with a standard mean difference of 1.19 ng/ml (95% CI: 0.59-1.70) (45). A second meta-analysis examined the correlation between circulating leptin and disease activity. This analysis concluded that circulating leptin levels are higher in patients with RA and that a small but positive correlation between leptin levels and parameters of disease activity, both DAS28 and CRP, exists (46). A correlation between circulating leptin and IL-17 has also been reported in RA (47). Finally, the role leptin may play on joint erosions in RA is another relevant question. One study reported that serum leptin was higher in patients with erosive disease, compared to those with non-erosive RA (48). In a study evaluating leptin in paired synovial and blood samples, concentrations of leptin were lower in synovial fluid than in plasma in patients with non-erosive RA, but not in patients with erosive RA (39). It was proposed that local consumption of leptin into the joint cavity may have a protective effect against erosion. Different studies analyzed the relationships between serum leptin and radiographic joint damage in RA, giving contradictory results (49–53). In three studies, serum leptin was not correlated with joint damage (51–53) while two other studies found that serum leptin levels were correlated with radiographic joint score: an inverse relationship between leptin levels and Larsen score was found in one study (Odds Ratio (OR): 0.32 [95% CI: 0.17-0.62]) (49) while in the second, progression of radiographic joint score was independently associated with serum leptin levels (OR: 1.59 [95% CI: 1.05- 2.42]) (50). Collectively, the question of the potential role of leptin on joint damage in RA is thus not clearly elucidated. Finally, leptin is included in the multi-biomarker disease activity (MBDA) score, a score calculated using an algorithm that includes the concentrations of 12 serum proteins playing a role in inflammatory and destructive processes. MBDA has been shown to be a strong predictor of radiographic damage (54).

### Adiponectin

Numerous studies demonstrated that adiponectin levels are increased in the serum but also in the synovial fluid of patients with RA, compared to healthy controls or patients with OA (42, 44, 49, 51–53, 55–57). Some, but not all studies found that adiponectin levels correlated with disease activity, CRP or DAS28 (42, 57). Adiponectin and its receptors AdipoR1 and AdipoR2 were found to be expressed in the synovial membrane of RA patients, especially by fibroblast synoviocytes (58). In addition, in an experimental model, adiponectin alone or in combination with IL-1β, is capable of inducing the release of IL-6, IL-8 and PGE2 by fibroblast synoviocytes (59). Adiponectin has catabolic properties on the cartilage (30) and may help osteoclastic activation through the stimulation of RANKL and the inhibition of osteoprotegerin production by osteoblasts, thus favoring cartilage and bone damage (12). Therefore, the potential of adiponectin in structural damage in RA has been examined in different studies. Collectively, it was reported that adiponectin is associated with the erosive process (49–53). Finally, in a Swedish cohort of obese subjects, elevated levels of adiponectin were associated with the development of RA, independently of CRP and levels of smoking (hazard ratio: 1.7 [95% CI: 1.12-2.6]) (60).

### Visfatin

Higher levels of serum visfatin were found in patients with RA, compared to healthy controls or patients with OA and a positive correlation between visfatin and measurements of disease activity has been described (42). A meta-analysis of circulating visfatin in RA concluded that visfatin levels are higher in RA than in controls and that there is a positive correlation between circulating levels and RA activity as evaluated by CRP or DAS28 (61). Visfatin has a catabolic function on cartilage by promoting the production of deleterious factors for the cartilage matrix components (25). In an animal model of arthritis, visfatin is required for osteoclastogenesis, suggesting a role in joint damage (62, 63). This was suggested by one study, which reported that visfatin concentrations were correlated with the radiographic Larsen score, independently of age, sex, disease duration, BMI and inflammation (OR: 2.38 [95% CI: 1.32- 4.29]) (49).

### Resistin

Resistin is mildly elevated in the serum of patients with RA while its concentration is markedly increased in the synovial fluid compared to results obtained from subjects with OA (35, 64). A meta-analysis of eight studies in RA showed that serum resistin is higher in patients with RA compared to the normal controls (65). In addition, resistin has been implicated in cartilage degradation by favoring the release of pro-inflammatory mediators and metalloproteases (25).

### Adipokines in Spondyloarthritis

**Figure 1:**
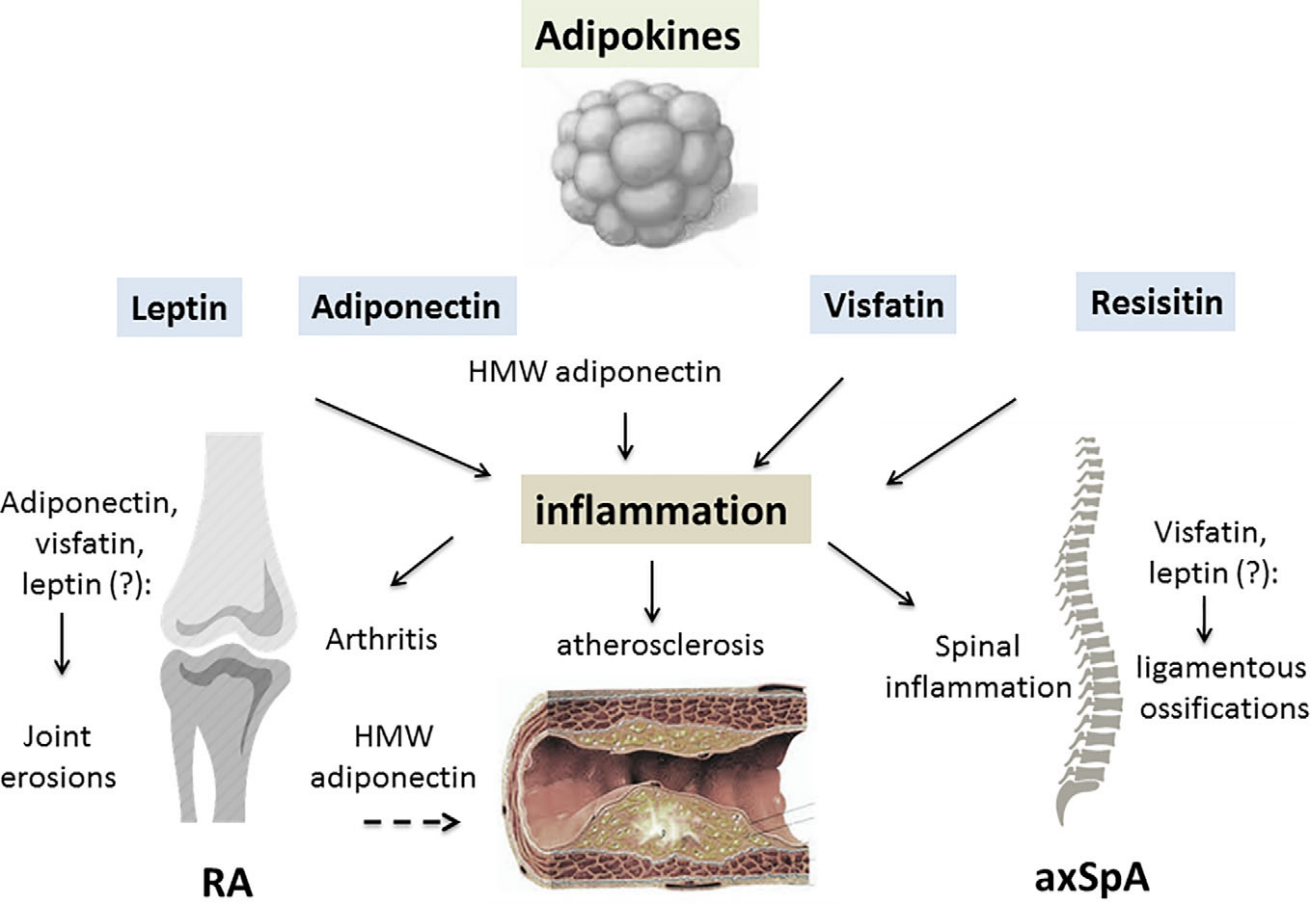
The contributing role of adipokines in inflammatory rheumatic diseases (IRD). A variety of factors are produced by white adipose tissue, collectively called adipokines. An excess of adipose tissue is described in patients with IRD, especially in rheumatoid arthritis (RA) and psoriatic arthritis (PsA). Leptin, adiponectin, visfatin and resistin may all be involved in the joint/axial skeleton inflammation of RA/spondyloarthritis (SpA) and PsA. By inducing pro-inflammatory cytokine release from innate and adaptive immune cells, adipokines generate an inflammatory environment. Leptin, visfatin and resistin all have pro-inflammatory effects on the joints and axial skeleton, while the consequences of adiponectin on the inflammatory process depend on its isoform. High molecular weight (HMW) adiponectin is thought to have mainly pro-inflammatory effects on joint structures. Due to interrelations the adipokines have with specific factors involved in cartilage and bone regulation, they may contribute to joint damage in RA or spinal ossification in SpA. Adipokines display metabolic properties such as regulation of glucose metabolism, especially for adiponectin, resistin and visfatin. IRD are characterized by a high prevalence of cardiometabolic complications, especially insulin resistance, metabolic syndrome and atherosclerosis. While leptin may have a negative influence on all these complications, HMW adiponectin may protect against the development of atherosclerosis in IRD (RA, rheumatoid arthritis; axSpA, axial spondyloarthritis; HMW, high molecular weight; dashed arrow, inhibitory effect; lined arrow, stimulating effect).

More limited information is available on adipokines in the SpA group. Circulating leptin levels have been reported be decreased in some studies (66, 67), but increased in another (68). Serum leptin correlated with measurements of disease activity (Bath ankylosing spondylitis disease activity index [BASDAI]) or acute phase reactants (CRP, IL-6) (69). A recent meta-analysis found no differences in serum leptin or serum adiponectin between AS and controls, while patients with AS had higher serum resistin levels (70). A second meta-analysis confirmed these results for serum leptin, indicating that there is no significant difference in plasma/serum leptin between patients and controls (71). In parallel, the question of the potential influence of adipokines on the progression of spinal ossifications has consistently been examined. Certain studies reported that serum leptin was more elevated in patients with syndesmophytes compared to patients without (72, 73) and one study reported the association between the changes in serum leptin at 2 years and radiographic progression (74). In parallel, visfatin but not resistin or adiponectin have been linked to worsening of the radiographic spinal ossification score (modified stoke ankylosing spondylitis spinal score [mSASSS]) (75). However, contradictory results have been reported, indicating a protective effect of leptin, together with HMW adiponectin, on spinal radiographic progression (**Figure 1**) (76).

### Adipokines in Psoriatic Arthritis

Contrary to psoriasis (77), adipokines have been less widely studied in PsA. One study reported higher serum levels of leptin and omentin, a secretory protein produced in visceral adipose tissue, and decreased levels of adiponectin in patients with PsA compared to healthy controls (78). Adipokines were compared between patients with psoriasis alone and patients with PsA in a large Canadian series, showing higher adiponectin levels in the PsA group and higher levels of leptin in PsA, but only among women. However, there was no control group in that study (79). Finally, higher circulating leptin, adiponectin and resistin levels have been reported in PsA compared to a group of healthy subjects (80). The relationship between these adipokines and disease activity was not clear, either with the acute phase reactants or with the clinical index of disease activity of PsA (80). Moreover, there is currently no evidence for a role of the adipokines leptin, adiponectin or others on structural joint damage in patients with PsA (78).

### Adipokines, Inflammatory Rheumatic Diseases and Cardiometabolic Complications

Besides these pro/anti-inflammatory biological effects, adipokines are involved in other functions that are relevant for IRD. Indeed, IRD are associated with cardiometabolic complications, and both leptin and adiponectin display metabolic properties. In RA, it has been shown that the leptin/adiponectin ratio, together with age and homeostasis model assessment of insulin resistance (HOMA-IR) index, is associated with carotid resistive index, an index of atherosclerosis severity (81). Leptin serum levels were independently related to CV events in patients with RA followed for a median of 40 months in a Chinese population (82). Furthermore, the Chinese study concluded that leptin may be considered as an independent prognostic factor for CV events in patients with RA (hazard ratio: 2.467 [95%CI 2.019–4.495]). Hypoadiponectinemia has been identified as a strong risk factor for CV disease (83). In addition, the cardiometabolic protective effects of adiponectin have been more closely related to its HMW isoform than total adiponectin in diabetic subjects (84). While the relationships between CV events, and adiponectin and its different isoforms have been extensively studied in the general population (85), this question has not been specifically examined in RA. However, based on results in subjects with traditional CV risk factors, it is assumed that adiponectin, especially its HMW isoform, plays a protective role in patients with RA (26).

## Conclusion

Taking account of the prevalence of obesity in IRD, available results on adipokines in IRD strongly support the posit that they play a contributing role in the systemic and local joint inflammation, especially RA. The relationships between adipokines and measurement of disease activity are not clearly demonstrated for each IRD, but highly suggested. A potential role of adiponectin in structural damage in RA has been suggested in certain studies. The current research gap includes the determination of the respective role the adipokines may play in arthritis, compared to cytokines, chemokines and other inflammatory mediators. In addition, a large number of other adipokines are described, for instance omentin, vaspin, chemerin, lipocalin-2, progranulin, retinol-binding protein-4, and they may all be involved in the inflammatory process and/or cardiometabolic regulation in IRD.

## Author Contributions

The author confirms being the sole contributor of this work and has approved it for publication.

## Funding

This work was supported by a grant from APICHU, CHU de Besançon, Besançon France.

## Conflict of Interest

The author declares that the research was conducted in the absence of any commercial or financial relationships that could be construed as a potential conflict of interest.
